# Serum Resistin Levels and Related Genetic Variants Are Associated With Bone Mineral Density in Postmenopausal Women

**DOI:** 10.3389/fendo.2022.868120

**Published:** 2022-08-05

**Authors:** Sundus Tariq, Saba Tariq, Saba Khaliq, Khalid Parvez Lone

**Affiliations:** ^1^ Physiology, University Medical & Dental College, The University of Faisalabad, Faisalabad, Pakistan; ^2^ Physiology and Cell Biology, University of Health Sciences, Lahore, Pakistan; ^3^ Pharmacology and Therapeutics, University Medical & Dental College, The University of Faisalabad, Faisalabad, Pakistan; ^4^ Pharmacology and Therapeutics, University of Health Sciences, Lahore, Pakistan; ^5^ Physiology/Metabolic Disorders, Government College University, Lahore, Pakistan

**Keywords:** osteoporosis, adipokine, resistin, single nucleotide variants, DXA (Dual-energy X-ray Absorptiometry)

## Abstract

**Background:**

Osteoporosis is a multifactorial disorder and a number of genetic variants or loci responsible for bone mineral density (BMD) have been identified. Resistin, a novel adipokine has diverse role in human body including its function in bone remodeling. The objective of this study was to see the association of serum resistin levels and related genetic variants (rs3931020, rs13144478) with BMD in postmenopausal females.

**Methods:**

This comparative analytical study was conducted on postmenopausal osteoporotic (n=101), osteopenic (n=77) and non-osteoporotic (n=74) females. For comparison and correlational analysis, Kruskal-Wallis test and Spearman’s rho correlation were used respectively. Hardy-Weinberg equilibrium (HWE) was calculated by using Chi-square test (χ^2^).

**Results:**

There was significant difference in the serum levels of resistin (*p <*0.001), among the three groups. Significant negative correlation of resistin was observed with BMD at various sites. Serum resistin levels were significantly low in the rs3931020 AA homozygous genotype (*p* = 0.010), and significantly high in the rs13144478 AT heterozygous genotype (*p* = 0.020), BMD at all sites except left femoral neck was significantly high in rs3931020 AA genotype, while BMD at lumbar spine, left hip and total BMD were significantly low in the rs13144478 TT homozygotes.

**Conclusion:**

High serum resistin levels are associated with low BMD and single nucleotide variation in rs3931020 and rs13144478 may lead to high serum resistin levels and low bone mineral density. Resistin can serve as a new genetic marker, potential therapeutic target and predictor of osteoporosis.

## Introduction

Resistin (RETN) is a low molecular weight (12.5 kDa) compound. It is a pre-polypeptide precursor that is composed of 108 amino acids in humans (1). Two different confirmations have been identified for human resistin, an oligomer and a trimer with a molecular weight of 660 kDa and 45 kDa respectively. Both forms are biologically active (2). The suggested receptors responsible for the effects of resistin are endotoxin receptor toll-like receptor 4 (TLR4), insulin growth factor-1 receptor (IGF-1R), tyrosine kinase-like orphan receptor-1 (ROR-1) and the adenylyl cyclase-associated protein 1 (CAP1) (3–6).

Resistin is highly expressed in the monocytes, macrophages and bone marrow cells, while small amounts are expressed by the hypothalamus, pituitary gland, spleen, pancreas, colonic epithelial cells, goblet cells, adrenal glands, adipose tissue, skeletal muscles, placental trophoblastic cells, and synovial tissue (2, 7).

Resistin has a diverse role in the human body and influence cellular structure and function of various tissues. It has pro-inflammatory properties and stimulates the production and secretion of pro-inflammatory cytokines (8) resulting in abnormal endothelial function (9).It generates oxidative stress and induces the proliferation of vascular smooth muscle cells (VSMCs) thus reducing the levels of nitric oxide (NO) resulting in reduced vasodilation, increased cell adhesion, thrombosis, angiogenesis and vascular permeability ultimately leading to atherosclerosis and increase incidence of cardiovascular events (10).

### Resistin and Bone

Resistin regulates the BMD (11) and is expressed by the murine pre-osteoclasts and pre-osteoblasts. In humans, its expression is seen in bone marrow stem cells and mature osteoblasts. It stimulates the proliferation of the osteoblasts, differentiation of the osteoclasts and release of various cytokines (1).

Resistin increases bone resorption by directly increasing the osteoclastogenesis resulting in the formation of biologically, active osteoclasts from the human monocytes that are capable of bone resorption, though it does not directly increase the activity of osteoclasts. It exerts an indirect stimulatory effect on the differentiation of the osteoclasts by increasing the synthesis and release of the IL-6, TNF-α, and PGE2 from the human peripheral monocytic cells. It has also an indirect weak inhibitory effect on osteoclastogenesis by reducing the RANKL/OPG mRNA ratio (12, 13).

Literature has shown equivocal relation of serum resistin levels with BMD and fractures at various sites. In a study conducted on postmenopausal females, there was negative correlation of serum resistin with BMD and it was found to be negative predictor of BMD (14). A study conducted on postmenopausal osteoporotic women has shown no association of serum resistin levels with BMD (15). Similarly, a study performed on subjects with osteoarthritis showed no convincing data to favor its association with BMD (16). In a recent study, conducted on pre and post-menopausal females, resistin was not found to be a predictor of BMD, and the association between fat mass and BMD was not found to be mediated by adipokines including resistin irrespective of their body composition and menopausal status (17). In another study, no association of resistin was found between postmenopausal normal, osteopenic and osteoporotic females (18).

### Single Nucleotide Variation in Resistin RETN Gene

Genetic variations may lead to disease development and can affect its course, progression, response to treatment and prognosis. Circulating resistin levels may be affected by the genetic factors. Resistin is encoded by *RETN* gene that is located on 19p13.2 (19). Genome wide association study (GWAS) has showed that serum levels of resistin were significantly associated with, *RETN* gene (19p13.2), and two novel loci located near the *NDST4* gene (4q25) and *TYW3/CRYZ* gene (1p31). These two novel loci were best represented by the SNP rs13144478 and SNP rs3931020, respectively. These new loci were also associated with *RETN* mRNA levels in white blood cells of type 2 diabetics. *TYW3/CRYZ* SNP rs3931020 also showed association with increased risk of coronary heart disease (20). As literature has shown the relation of serum resistin levels with BMD, So, association of these SNPs (rs13144478 and rs3931020) from two novel loci has been evaluated with BMD in this study. The location of rs13144478 is on chr4:115275150 (GRCh38.p12) and position of rs3931020 is on chr1:74769633 (GRCh38.p12), until date, no clinical significance has been reported in ClinVar for both of them.

The objective of the study was to see the association of serum resistin levels and related genetic variants (rs3931020, rs13144478) with bone mineral density in postmenopausal women.

## Materials and Methods

This comparative analytical study was conducted over a period of three years and included 252 postmenopausal women, divided into three groups, non-osteoporotic (n = 74), osteopenic (n = 77) and osteoporotic (n = 101). Ethical review board of University of Health Sciences, Lahore has given approval to conduct this study in accordance with Helsinki declaration of human rights (21).

Initially, a bone density screening camp was organized in outpatient department of Madina Teaching Hospital, Faisalabad, Pakistan, which is a tertiary care hospital with all the necessary facilities to carry out this research. Postmenopausal females were invited for screening using quantitative ultrasound scan. BMD was assessed from the calcaneus for the purpose of screening. Twenty-five hundred postmenopausal females were screened and interviewed by the doctor after obtaining written informed consent. General information including age, marital status, menstrual history, past medical, surgical and drug history was obtained. Postmenopausal women between 50 to 70 years of age and minimum 2 years of amenorrhea were included while women on medication affecting bone mineralization, taking vitamin D or bisphosphonate therapy, with chronic liver or renal disease, malignancies, autoimmune diseases, endocrine (diabetes mellitus) or parathyroid hormone problems, iatrogenic and premature menopause were excluded from the study. The screened subjects, fulfilling the inclusion and exclusion criteria were sent for dual energy X-ray absorptiometry (DXA) analysis.

### Estimation of Bone Mineral Density

Bone mineral density (BMD) of postmenopausal females was evaluated at the lumbar spine (L2-L4), right femoral neck, right hip, left femoral neck and left hip by dual energy X-ray absorptiometry (DXA) using HOLOGIC-HORIZON (QDR-series), dual energy X-ray absorptiometry system. The results of DXA were used for final analysis and presented as T-score. According to the criteria set by world health organization (WHO), osteoporosis in adults is diagnosed by the T-scores obtained from DXA. T-score is defined as, the comparison of measured BMD result with the average BMD of the young adults at the time of peak bone mass. T-score ≤ 2.5 standard deviations below the mean peak bone mass represent osteoporosis (22). Postmenopausal non-osteoporotic women (n=74) having T-score ≥ -1.0, osteopenic women (n=77) with T-score between -1.0 to -2.5 and osteoporotic women (n=101) with T-score ≤ -2.5 were finally included in the study for analysis.

### Biochemical Analysis

Blood samples (6 mL) were obtained after overnight fasting. Two mL blood was collected in ethylene diamine tetraacetate (EDTA)-vacutainer and stored at -40 °C for DNA extraction. Serum was extracted from four mL blood after centrifugation at 3000 revolutions per minute or 1000xg for 10min. Serum resistin levels were quantified by human resistin enzyme linked immunosorbent assay (ELISA) formulated by Elab science Biotechnology Inc. with a sensitivity of 18.75 pg/mL, coefficient of variation <10% and almost nil cross reactivity. The intra-assay and inter-assay coefficient of variation for low, middle and high levels of serum resistin was 6.79%, 5.61%, 4.6% and 6.88%, 5.61%, 3.7% respectively. The assay range of the kit was 31.25-2000 pg/mL. Samples were initially run with serial dilutions and a sample dilution of 1:10 was finalized which was used to quantify the results. The final values were presented in ng/mL. The biochemical analysis was performed using microplate data collection and analysis software Gen5TM and Gen5 Secure, manufactured by BioTekVR Instruments, Inc.

### Detection of Polymorphisms and Genetic Variations

DNA from whole blood was extracted using GeneJet whole blood genomic DNA purification mini kit, manufactured by Thermo Fisher Scientific Inc. Carlsbad, California 92008, USA.

The genetic variants were selected, after thorough literature survey and Ensemble search, from selected genes and nearby loci, which are suspected to be involved in its regulation. Primers were designed, by using Tetra-primer ARMS-PCR tool (http://primer1.soton.ac.uk/primer1), and Primer3Plus tool (http://www.bioinformatics.nl/cgi-bin/primer3plus/primer3plus.cgi). All the designed primers were then BLAST in the bioinformatics tool (www.basic.northwestern.edu/biotools/oligocalc.html) to determine homology and avoid mismatch and secondary structure formation within the primers. Primer sequences (5’ - 3’) for selected genetic variants are as below.

**TYW3/CRYZ (loci of RETN)**

rs3931020-F1 CTAGGCAAGTGCCAATACAAACACAA

rs3931020-F2 TTTCCTTCTAGTAACATTATTAAATAC

rs3931020-R1 TGTGGTTATGTAAGTATAAACAAAATGC

rs3931020-R2 AGAAAGTGAACTATTTCTCAATAACCAG

**NDST4 (loci of RETN)**

rs13144478-F1 TGAATATATTTTGAAAATGAATGCATGAT

rs13144478-F2 GAAAGCCAAAGGAGTTCCATATACAT

rs13144478-R1 TGATTTTTAAATCACTTTGCATATGTGA

rs13144478-R2 GTATGTATGGAAACCAGCAGGTTATTTA

The genetic regions of different genes were amplified from extracted DNA using gene specific primers under optimized conditions. After PCR amplifications, analysis of genetic variants rs3931020 and rs13144478 were done by amplification refractory mutation system (ARMS)-PCR. Amplicons were checked using agarose gel electrophoresis. For rs3931020 variant, 75 samples and for rs13144478 variant, 24 samples could not be genotyped or they yielded ambiguous results, so they were excluded from the analysis. The final sample included for analysis for rs3931020 variant was 177 and for rs13144478 variant was 228.

### Sequencing

The results of PCR for selected single nucleotide variants were confirmed by sequencing. Samples were sequenced by Advance Bioscience International (ABI) China. The data obtained after sequencing was then visualized by using Chromas software and Sequencher^®^5.4.6 to find out the variations in these sequences.

### Statistical Analysis

Data were entered and analyzed using SPSS version 26.0 (Statistical Package for Social Sciences). Distribution of the data was checked by Shapiro-Wilk’s statistics and if p-value was < 0.05, data was considered to be non-normally distributed. Mean ± SD (Standard deviation) was given for normally distributed quantitative variables. Median with IQR (Interquartile range) was given for non-normally distributed quantitative variables. Frequencies and percentages were given for categorical variables. Proportions and percentages were compared using Chi-square test. Kruskal-Wallis test was applied to compare non-normally distributed quantitative variables among the three groups. *Post-hoc* pairwise comparisons were performed using Dunn-Bonferroni approach. Spearman’s Rho correlation was applied to observe correlations between non-normally distributed quantitative variables.

Genotypic and allelic frequencies were calculated. In order to study the frequency and association of polymorphisms with the study groups, three genetic models (Co-dominant Model, Dominant Model, and Recessive Model) were constructed (23). Genotype frequencies of three groups were compared by Chi-square test (χ^2^). Association of various biochemical markers and BMD with genotype frequencies were observed using Kruskal-Wallis test. P-value ≤ 0.05 was taken as statistically significant.

## Results

The study included 252 postmenopausal women, divided into three groups, non-osteoporotic (n = 74), osteopenic (n = 77) and osteoporotic (n = 101). The general characteristics and serum resistin levels are given in **Table 1**. The study population was age matched, as the median age between the groups was statistically non-significant (*p* = 0.063). There was significant difference in the serum levels of resistin (*p <*0.001), among the three groups. Multiple comparisons after Dunn-Bonferroni correction showed that serum resistin levels were significantly low in non-osteoporotic postmenopausal females as compared to osteopenic (*p <*0.001) and osteoporotic females (*p <*0.001).

**Table 1:** General characteristics of the study population.

Parameters		Normal^x^ n = 74	Osteopenic^y^ n = 77	Osteoporotic^z^ n = 101	*p*-value*
Age (years) median (IQR)^a^		56 (50-64)	57 (54-63)	57 (53-65)	0.063
Menopausal age (years) median (IQR)^a^		50 (48-54)	50 (46-53)	50 (48-52)	0.084
Marital status n (%)^b^	Married	74 (100)	77 (100)	99 (98)	0.222
	Unmarried	0 (0)	0 (0)	2 (2)	
Smoking n (%)^b^	No	61 (82.4)	65 (84.4)	77 (76.2)	0.072
	Active	4 (5.4)	9 (11.7)	17 (16.8)	
	Passive	9 (12.2)	3 (3.9)	7 (6.9)	
History of fracture n (%)^b^	Yes	6 (8.1)	9 (11.7)	20 (19.8)	0.070
	No	68 (91.9)	68 (88.3)	81 (80.2)	
Resistin (ng/mL) median (IQR)^a^		2.04 (0.68-4.71)	5.33 (1.93-10.83)	6.77 (2.14-12.36)	<0.001*

*p-value ≤ 0.05 is considered statistically significant.

Comparisons are seen using Kruskal–Wallis test^a^ and Chi-square test^b^.

Normal^x^, represents the postmenopausal non-osteoporotic women; Osteopenic^y^, represents the postmenopausal osteopenic women and Osteoporotic^z^, represents the postmenopausal osteoporotic women group.

**Figure 1:**
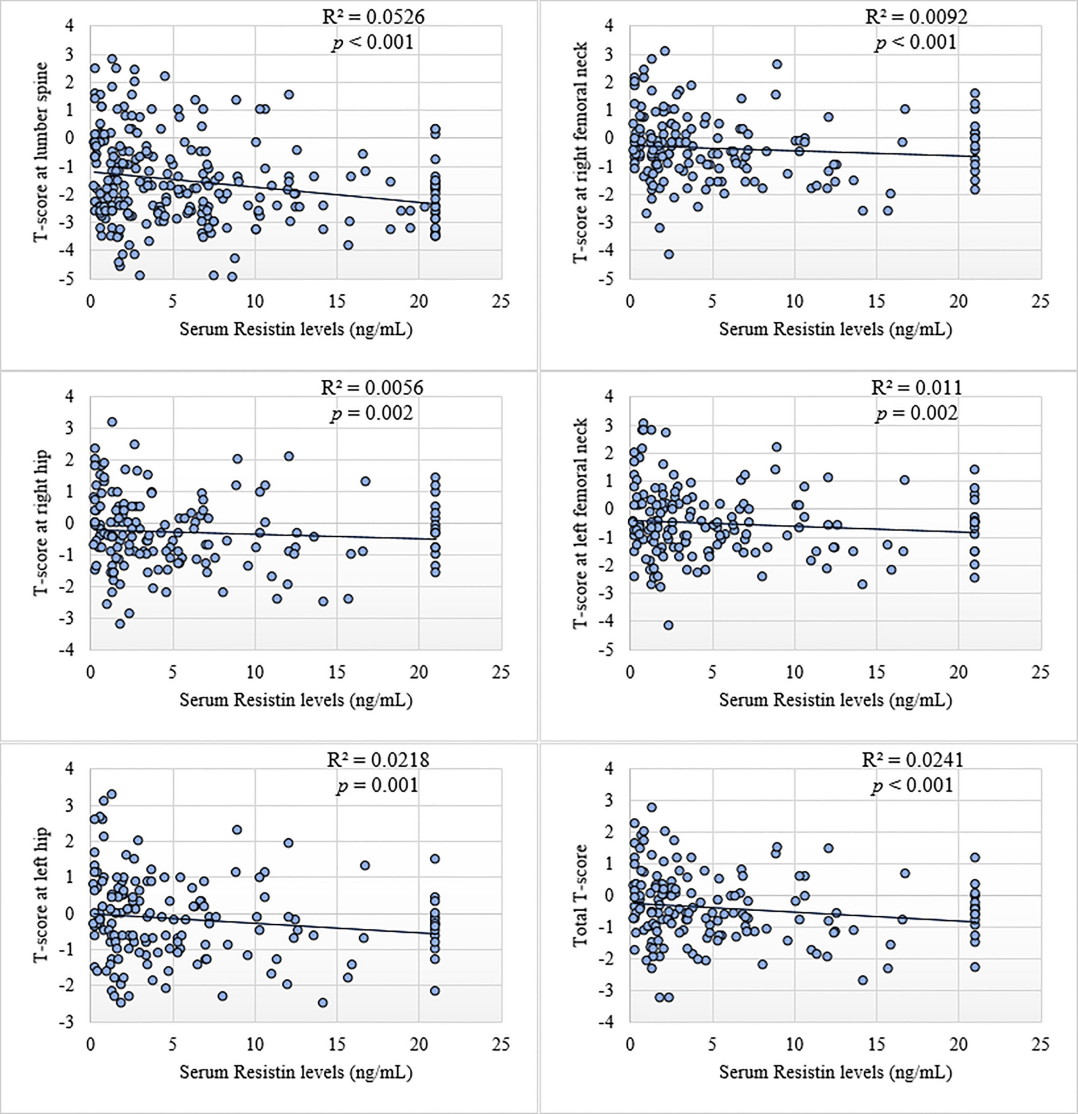
Scatter plots showing correlation of BMD (T-scores) with serum resistin levels in postmenopausal women.

On correlating serum resistin levels with BMD, it was observed that there is highly significant, negative correlation of serum resistin levels with BMD (**Figure 1**). After adjusting for age, menopausal age, BMI and smoking status, this correlation remained significant at lumbar spine (rho = -0.211, *p* = 0.001), right femoral neck (rho = -0.148, *p* = 0.020), right hip (rho = -0.131, *p* = 0.039), left femoral neck (rho = -0.153, *p* = 0.016), left hip (rho = -0.177, *p* = 0.005) and total BMD (rho = -0.184, *p* = 0.004).

### rs3931020 Variant in Study Groups

**Figure 2:**
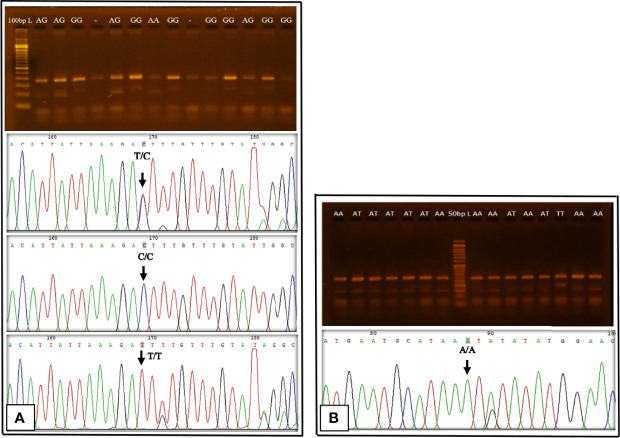
Gel electrophoresis pattern of ARMS-PCR and Sequence chromatographs showing rs3931020 and rs13144478 variants. **(A)**: rs3931020, DNA ladder = 100 base pairs (bp), PCR product size for A allele =192 bp, G allele = 229 bp, two outer primers = 368 bp; Sequence chromatograph of rs3931020 is reverse complement. **(B)**: rs13144478, DNA ladder = 50 bp, PCR product size for A allele =148 bp, T allele = 189 bp, two outer primers = 282 bp.

The electrophoresis pattern of ARMS-PCR for detection of rs3931020 variant is shown in **Figure 2**. PCR product sizes were 192 bp for A allele, 229 bp for G allele, and 368 bp for two outer primers. Sequence chromatographs showing rs3931020 variants are shown in **Figure 2**. The allelic and genotypic frequencies of rs3931020 variant of the novel loci of resistin are shown in **Table 2**.

**Table 2:** Comparison of rs3931020 and rs13144478 genotypic and allelic frequencies among the study groups.

	rs3931020 variant					rs13144478 variant				
Models	Genotypes	Normaln (%)	Osteopenicn (%)	Osteoporoticn (%)	p-value	Genotypes	Normaln (%)	Osteopenicn (%)	Osteoporoticn (%)	p-value
Co-dominant	AA	21 (39.6)	9 (18.4)	12 (16.0)	0.017*	AA	49 (72.1)	34 (51.5)	48 (51.5)	0.050*
	AG	15 (28.3)	24 (49.0)	32 (42.7)		AT	11 (16.2)	16 (24.2)	28 (29.8)	
	GG	17 (32.1)	16 (32.7)	31 (41.3)		TT	8 (11.8)	16 (24.2)	18 (19.1)	
	Total	53 (100)	49 (100)	75 (100)		Total	68 (100)	66 (100)	94 (100)	
Allelic	A	57 (53.8)	42 (42.9)	56 (37.3)	0.032*	A	109 (80.1)	84 (63.6)	124 (66.0)	0.005*
	G	49 (46.2)	56 (57.1)	94 (62.7)		T	27 (19.9)	48 (36.4)	64 (34.0)	
	Total	106 (100)	98 (100)	150 (100)		Total	136 (100)	132 (100)	188 (100)	
Dominant	AA	21 (39.6)	9 (18.4)	12 (16.0)	0.005*	AA	49 (72.1)	34 (51.5)	48 (51.5)	0.015*
	AG+GG	32 (60.4)	40 (81.6)	63 (84.0)		AT+TT	19 (27.9)	32 (48.5)	46 (48.9)	
	Total	53 (100)	49 (100)	75 (100)		Total	68 (100)	66 (100)	94 (100)	
Recessive	GG	17 (32.1)	16 (32.7)	31 (41.3)	0.469	TT	8 (11.8)	16 (24.2)	18 (19.1)	0.171
	AA+AG	36 (67.9)	33 (67.3)	44 (58.7)		AA+AT	60 (88.2)	50 (75.8)	76 (80.9)	
	Total	53 (100)	49 (100)	75 (100)		Total	68 (100)	66 (100)	94 (100)	

Chi square test was applied to compare frequencies.

*p-value ≤ 0.05 is considered statistically significant.

The χ^2^ test of rs3931020 variants in the studied subjects suggested that the variant sites does not corresponded to Hardy–Weinberg equilibrium (*p* < 0.05). Significant differences were observed in genotype frequencies between the groups concerning rs3931020 variants (χ^2 =^ 12.016, *p* = 0.017). In the dominant effect of the G allele (comparison between AG+GG vs. AA), significant association was found between the dominant model and study groups (χ^2^ = 10.651, *p* = 0.005). AG+GG genotypes may significantly increase the risk for both osteopenia and osteoporosis in the study population. By contrast, in the recessive effect of the G allele (comparison between GG vs. AA+AG), no significant association was found (χ^2^ = 1.514, *p* = 0.469). Significant association was also found between the allelic model and the study groups (χ^2^ = 6.868, *p* = 0.032). G allele may be associated with the risk of osteoporosis.

The results of association analysis of genotypes of rs3931020 polymorphism with serum resistin levels and BMD at various sites in the studied subjects showed a significant difference in the serum levels of resistin between different rs3931020 genotypes (*p* = 0.010) (**Table 3**), and remained significant between AA homozygotes and AG heterozygotes after Dunn-Bonferroni correction (*p* = 0.008).

**Table 3:** Serum resistin levels and BMD in postmenopausal women with rs3931020 and rs13144478 single nucleotide variation.

	rs3931020 variant				rs13144478 variant			
	AA n = 42	AG n = 71	GG n = 64	*p*-value	AA n = 131	AT n = 55	TT n = 42	*p*-value
Resistin	2.24^#^ (1.09 to 4.94)	5.87^#^ (1.86 to 11.32)	4.41 (1.54 to 9.94)	0.010*	3.56^#^ (1.28 to 7.23)	6.77^#^ (2.40 to 12.53)	3.61 (1.35 to 8.73)	0.020*
Lumbar spine	-0.95 (-2.42 to -0.10)	-1.90 (-2.70 to -0.90)	-1.90 (-2.70 to -0.42)	0.050*	-1.50^#^ (-2.60 to -0.20)	-2.00 (-2.70 to -1.30)	-2.05^#^ (-3.00 to -1.32)	0.008*
Right femoral neck	-0.25^#^ (-1.82 to 0.50)	-1.50^#^ (-2.50 to -0.30)	-0.95 (-2.22 to -0.22)	0.041*	-0.80 (-1.90 to 0.10)	-1.30 (-2.50 to -0.40)	-1.60 (-2.27 to -0.37)	0.017*
Right hip	-0.15^#^ (-1.60 to 0.82)	-1.10^#^ (-2.00 to -0.00)	-1.00 (-2.10 to 0.00)	0.022*	-0.80 (-1.60 to 0.40)	-1.00 (-2.10 to -0.40)	-1.40 (-2.20 to -0.27)	0.038*
Left femoral neck	-0.75 (-1.92 to 0.12)	-1.40 (-2.30 to -0.50)	-1.35 (-2.00 to 0.50)	0.163	-1.00 (-2.00 to 0.10)	-1.50 (-2.20 to -0.50)	-1.50 (-2.32 to -0.77)	0.031*
Left hip	-0.30^#^ (-1.23 to 0.82)	-1.00^#^ (-1.80 to -0.20)	-0.80 (-1.80 to 0.27)	0.018*	-0.60^#^ (-1.40 to 0.40)	-1.00 (-1.80 to 0.00)	-1.00^#^ (-2.12 to -0.27)	0.016*
Total BMD	-0.52^#^ (-1.83 to -0.30)	-1.50^#^ (-2.10 to -0.46)	-1.22 (-1.95 to -0.25)	0.034*	-0.80^#^ (-1.92 to 0.06)	-1.60 (-2.02 to -0.64)	-1.50^#^ (-2.34 to -0.62)	0.006*

Comparisons are seen using independent sample Kruskal-Wallis test.

Values are given in median (IQR).

Post-hoc comparisons were seen using the Dunn-Bonferroni approach.

^#^Post-hoc comparisons showed significance between AA - AG, AA - AT, and AA - TT.

*p-value ≤ 0.05 is considered statistically significant.

There was also significant difference in BMD at lumbar spine (*p* = 0.050), right femoral neck (*p* = 0.041), right hip (*p* = 0.022), left hip (*p* = 0.018) and total BMD (*p* = 0.034) between rs3931020 genotypes (**Table 3**).

Multiple comparisons after Dunn-Bonferroni correction showed that BMD at right femoral neck (*p* = 0.036), right hip (*p* = 0.028), left hip (*p* = 0.015) and total BMD (*p* = 0.033) was significantly low in the rs3931020 AG heterozygotes as compared to the rs3931020 AA homozygotes.

### rs13144478 Variant in Study Groups

The electrophoresis pattern of ARMS-PCR for detection of rs13144478 variant is shown in **Figure 2**. PCR product sizes were 148 bp for A allele, 189 bp for T allele, and 282 bp for two outer primers. Sequence chromatograph showing rs13144478 variant is shown in **Figure 2**. The allelic and genotypic frequencies of rs13144478 variant of the novel loci of resistin are shown in **Table 3**.

The χ^2^ test of rs13144478 variants in the studied subjects suggested that the variant sites does not corresponded to Hardy–Weinberg equilibrium (*p* < 0.05). Significant differences were observed in genotype frequencies between the groups concerning rs13144478 variants (χ^2 =^ 9.505, *p* = 0.050). In the dominant effect of the T allele (comparison between AT+TT vs. AA), significant association was found between the dominant model and study groups (χ^2^ = 8.456, *p* = 0.015). AT+TT genotypes may significantly increase the risk for both osteopenia and osteoporosis. By contrast, in the recessive effect of the T allele (comparison between TT vs. AA+AT), no significant association was found (χ^2^ = 3.526, *p* = 0.171). Significant association was also found between the allelic model and the study groups (χ^2^ = 10.530, *p* = 0.005). T allele may be associated with the risk of osteoporosis.

The results of association analysis of genotypes of rs13144478 polymorphism with serum resistin levels and BMD at various sites in the studied subjects showed significant difference in the serum levels of resistin between rs13144478 genotypes (*p* = 0.020) (**Table 3**), and remained significant between AA homozygotes and AT heterozygotes after Dunn-Bonferroni correction (*p* = 0.015).

There was also significant difference in BMD at lumbar spine (*p* = 0.008), right femoral neck (*p* = 0.017), right hip (*p* = 0.038), left femoral neck (*p* = 0.031), left hip (*p* = 0.016) and total BMD (*p* = 0.006) between rs13144478 genotypes (**Table 3**).

Multiple comparisons after Dunn-Bonferroni correction showed that BMD at lumbar spine (*p* = 0.025), left hip (*p* = 0.025) and total BMD (*p* = 0.016) was significantly low in the rs13144478 TT homozygotes as compared to rs13144478 AA homozygotes.

## Discussion

Osteoporosis is a highly prevalent condition especially in postmenopausal females that leads to development of fractures and disability.

In the present study, serum levels of resistin were significantly high in postmenopausal osteopenic and osteoporotic groups as compared to the non-osteoporotic females. Similar findings were observed in other study where relationship of various biochemical markers of bone metabolism with serum resistin levels in older patients was seen and a direct relation of serum resistin levels with cervical fracture was found that may be attributed to the inverse relation of resistin with osteocalcin (24). Resistin levels were also found to be higher in other diseases related to bone like ankylosing spondylitis (25). A study conducted on postmenopausal obese women from Tunisia has shown positive relation of resistin with bone resorption markers (C-terminal telopeptide, CTX-I), showing its probable role in bone remodeling (26).

As compared to the present study, contrasting results were observed in other studies. The studies conducted on Chinese subjects (16) and postmenopausal Polish women (15) have shown that serum resistin was not an independent predictor of BMD. While another study on male and female osteoporotic subjects has shown it to be inversely related to lumbar spine BMD (27). *In vitro* analysis showed that, in mesenchymal stem cells (MSC) and primary human bone marrow derived mesenchymal stromal cells (hMSC), matrix mineralization and expression of type 1 collagen was not altered by resistin neither the synthesis of cytokines during osteogenic or adipogenic differentiation (28).

The present study has also revealed significant negative correlation of serum resistin levels with bone mineral density at lumbar spine, right femoral neck, right hip, left femoral neck, left hip and total BMD. Though very limited data are available but *in vitro* studies has strongly supported the findings in this study. Resistin is expressed by many cells but highest expression has been seen in the osteoclasts at their earlier stages of differentiation. There was a two-fold increase in the differentiation of osteoclasts from human peripheral monocytes after 12th day of induction with 10 nM of resistin. Similarly, the expression of IL-6 mRNA and IL-6 release were also increased while that of receptor antagonist of nuclear factor κB ligand (RANKL) mRNA was weakly decreased in murine cells after treatment with resistin. *In vitro* studies have also shown an increase in osteoclastogenesis in response to recombinant resistin by inducing the maturation of osteoblasts from pre-osteoblasts (29).

**Figure 3:**
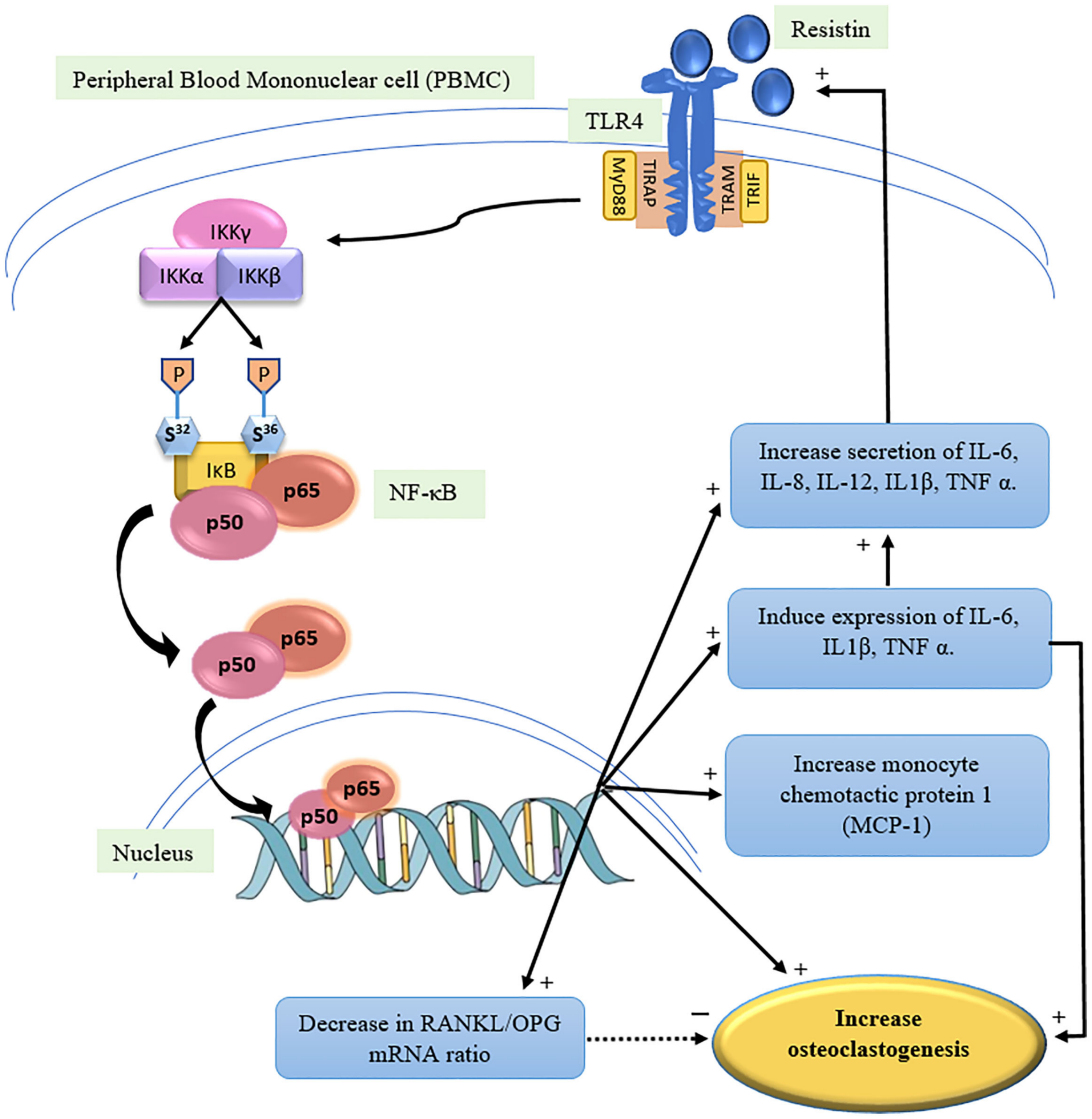
Mechanism of action of Resistin to increase osteoclastogenesis (proposed) (1, 3, 12, 26). TLR4: Toll like receptor, MyD88: Myeloid differentiation primary response gene 88, TIRAP: Toll/interleukin-1 receptor domain containing adaptor protein, TRIF: TIR-domain-containing adaptor inducing interferon-β, TRAM: TRIF-related adaptor molecule, IKK: IκB kinase complex, NF-κB: Nuclear factor kappa light chain enhancer of activated B cells, RANKL: receptor antagonist of nuclear factor κB ligand, OPG: Osteoprotegerin.

The NF-κB pathway plays a vital role in differentiation of osteoclasts. Pro-inflammatory role of resistin, increases IL-6, IL-1β, TNF-α, synthesis thus increasing osteoclastogenesis (28). The proposed mechanism of resistin causing decrease in BMD by directly and indirectly increasing osteoclastogenesis is shown in **Figure 3**. Binding of resistin to TLR4 receptor leads to its dimerization and activation of MyD88 dependent pathway. IκB keeps the NF-κB dimer in an inhibitory form inside the cytoplasm. After stimulation by the MyD88 dependent pathway, there is phosphorylation and further degradation of IκB through a ubiquitin/proteasomal process. This phosphorylation is mediated by the IKK complex (IKKγ, IKKα, IKKβ). Release of IκB activates the NF-κB, and exposes the nuclear localization subunits p50 and p65 (p50 is the DNA binding subunit and p65 is transactivator), allowing import into the nucleus and activation of transcription of target genes (30). Resistin also decreases the release of osteocalcin from osteoblasts (31). The carboxylated form of osteocalcin binds with calcium and concentrates it inside the bone thus contributing in mineralization of bone, it gets decarboxylated in low pH of osteoclast resorption compartment losing its calcium binding capacity and affinity with bone tissue (32).

This study has also investigated the possible association of novel loci of resistin represented by SNPs rs13144478 and rs3931020 with BMD. This is the first study to investigate such an association. Beckers et al. (2013) investigated the role of *RETN* variants (rs1862513, rs3745367 and rs3745369) in obesity and bone mineral density and were unable to find any association between these variants and bone related parameters (33).

In the present study, significant differences were observed in genotype frequencies between the groups concerning rs3931020 and rs13144478 polymorphism. The co-dominant and dominant tested inheritance models of rs3931020 and rs13144478 polymorphisms were significantly different between the three groups. In rs3931020, the A allele seems to be protective against osteoporosis and single nucleotide variations in rs3931020 may lead to the development of osteoporosis. In rs13144478, the T allele increased the risk of osteoporosis.

In Pakistan, the marriages are inter racial most of the times and the locals prefer to get married with in their own castes. Disequilibrium can arise from population substructure or inbreeding (23), rather than genotyping error.

It is also well known that males receive their X chromosome from their mothers, male allele frequencies of X-chromosomal markers equal the female allele frequencies of the previous generation. If male and female allele frequencies initially differ, then it will take several generations before HWE is reached. This shows that the inclusion of the males can drastically change the statistical inference on HWE. Inclusion of the males also lessens the evidence for disequilibrium to some extent, bringing the *p*-value above the 5% threshold (34). As in this study, we have taken only female population this could be one of the reason of this disequilibrium.

The results of association analysis of genotypes of rs3931020 polymorphism with serum resistin levels and BMD at various sites in the studied subjects showed that serum resistin levels were significantly low in the rs3931020 AA genotype. BMD at all sites except left femoral neck was significantly high in rs3931020 AA genotype. The presence of rs3931020 AA genotype is associated with lower serum resistin levels and high BMD. Therefore, single nucleotide variation in rs3931020 may lead to high serum resistin levels and low bone mineral density that may further increase the risk of osteoporosis in postmenopausal females.

The results of association analysis of genotypes of rs13144478 polymorphism with serum resistin levels and BMD at various sites showed that serum resistin levels were significantly high in the rs13144478 AT heterozygous genotype while BMD at lumbar spine, left hip and total BMD were significantly low in the TT homozygotes. Therefore, single nucleotide variation in rs13144478 may lead to high serum resistin levels and low bone mineral density that may further increase the risk of osteoporosis in postmenopausal females. No comparative data was available making these variants novel.

## Conclusion

The results conjecture that serum resistin levels are associated with BMD and rs3931020 and rs13144478 polymorphisms could be used together with other genetic markers to identify postmenopausal females at higher risk of developing osteoporosis. Single nucleotide variations in rs3931020 and rs13144478 may act as a potential biomarker for osteoporosis screening, diagnosis and future treatment.

## Data Availability Statement

The original contributions presented in the study are included in the article/supplementary materials. Further inquiries can be directed to the corresponding authors.

## Ethics Statement

The study was approved by Institutional Review Board, University of Health Sciences, Lahore. The patients/participants provided their written informed consent to participate in this study.

## Author Contributions

SuT: Conception and design, acquisition, analysis, interpretation of data, drafted the manuscript. SaT: Acquisition, analysis of data, carried out the literature search, helped in drafting the manuscript. SK: Designed and supervised the research, analysis and interpretation of data, revised the manuscript critically for important intellectual content. KL: Designed and supervised the research, revised the manuscript critically for important intellectual content. The final manuscript is approved by all authors for publication. All authors contributed to the article and approved the submitted version.

## Conflict of Interest

The authors declare that the research was conducted in the absence of any commercial or financial relationships that could be construed as a potential conflict of interest.

## Publisher’s Note

All claims expressed in this article are solely those of the authors and do not necessarily represent those of their affiliated organizations, or those of the publisher, the editors and the reviewers. Any product that may be evaluated in this article, or claim that may be made by its manufacturer, is not guaranteed or endorsed by the publisher.
